# Reconstruction With Peroneus Longus Allograft for Chronic Subtotal Patellar Tendon Lesion: Surgical Technique and Clinical Implications

**DOI:** 10.7759/cureus.97782

**Published:** 2025-11-25

**Authors:** Giovanni Longo, Massimo Cipolla, Aaron Alberto Guzman Tello, Jessica Nicole Ordoñez Reyna, Carmine Zoccali

**Affiliations:** 1 Orthopaedics and Traumatology, Sapienza University of Rome, Rome, ITA

**Keywords:** allograft, chronic tendon injury, extensor mechanism, peroneus longus tendon graft, pt reconstruction

## Abstract

Chronic patellar tendon (PT) injuries lead to anterior knee pain and loss of active extension, often requiring reconstruction when direct repair is not feasible. We present a case of a 67-year-old patient with a history of poliomyelitis and a previous patellar fracture, who developed a chronic subtotal PT rupture that had been left untreated. Reconstruction was performed using a peroneus longus tendon allograft. After debridement of degenerated tissue, two converging patellar bone tunnels and one transverse tibial tunnel were created. The graft was whipstitched at both ends, passed through the tunnels, and fixed to the tibial tuberosity with non-absorbable suture anchors. At six months, the patient showed substantial improvement in range of motion (-5°-110°), extensor strength (0/5→3/5), and pain (VAS 7→4), along with better functional scores (KSS 45→65; Tegner 1→2-3). At 12 months, the reconstruction remained stable with further recovery. The peroneus longus allograft provided adequate strength, suitable dimensions, and no donor-site morbidity, representing a reliable option for chronic PT reconstruction in patients with poor local tissue quality or comorbidities.

## Introduction

Patellar tendon (PT) rupture is a rare but highly disabling condition, characterized by acute pain, a palpable discontinuity, and the inability to actively extend the knee. This injury severely compromises the extensor mechanism, which is essential for both daily activities and sports performance. Surgical management represents the standard of care, since conservative treatment inevitably leads to tendon retraction, biomechanical alterations, and poor long-term functional outcomes [1,2].

Specific risk factors commonly associated with PT rupture include corticosteroid use, diabetes mellitus, chronic kidney disease, and systemic lupus erythematosus. However, these injuries more frequently result from trauma or sports-related accidents [3] or may occur as a consequence of previous surgical repair of patellar fractures.

Acute ruptures generally benefit from direct repair, whereas chronic lesions, defined as those persisting for more than six weeks, pose greater surgical challenges due to proximal patellar migration, scarring, and poor quality of residual tissue, which often precludes direct suture [2,4]. In such cases, reconstruction using autografts, allografts, or synthetic materials becomes necessary to restore patellar height and knee extensor function [4,5].

Over the years, numerous techniques have been described; they can be classified based on the tissue used for reconstruction as autologous, homograft, or artificial graft.

Hamstring tendons (semitendinosus and gracilis), the Achilles tendon with or without a bone block, and the contralateral PT with or without a bone block are the most commonly used autologous tendons for ligament reconstruction.

They are characterized by excellent biomechanical properties and high biological potential for integration, but their use requires an additional surgical procedure for graft harvesting, which may be associated with donor-site morbidity (such as pain, weakness, sensory disturbances, hematoma, or even tendon rupture).

Homologous tendons, such as hamstrings, are also widely used for ligament reconstruction.

They eliminate the risk of donor-site complications, but they present other critical issues, including a higher risk of graft rejection, disease transmission, slower biological integration, and inferior biomechanical properties compared to fresh autografts. Moreover, sterilization and preservation processes can further reduce graft strength and increase the rate of late failures.

Artificial tendons are the least used option and are reserved for cases in which neither autologous nor homologous tendons are available.

Their indications are very limited because of poor long-term outcomes, mechanical failure, inflammatory reactions, and high complication rates.

Although autologous ligaments should be associated with the best results, available systematic reviews highlight the absence of a definitive consensus regarding the superior technique. Some authors suggest that autografts may provide an advantage, with lower complication and failure rates compared to isolated repair [2,6].

Other studies have reported favorable outcomes with both allograft and synthetic reconstructions, although significant heterogeneity in outcomes and generally low levels of evidence persist [4,7,8].

Given the rarity and complexity of chronic PT lesions, especially in neuromuscular patients, detailed case reports remain essential to guide surgical decision-making and improve awareness of alternative graft options.

In light of these considerations, this article describes a surgical technique for PT reconstruction following chronic rupture secondary to patellar cerclage wiring for fracture in a patient with a history of poliomyelitis.

## Technical report

A 67-year-old patient with a history of poliomyelitis and a previous patellar fracture treated with tension band wiring in 2017, subsequently removed in 2018, presented in 2019 with a PT rupture that was initially left untreated. Due to persistent extensor deficit, a clinical evaluation and magnetic resonance imaging (MRI) were performed, confirming a chronic subtotal rupture of the PT with proximal retraction.

The patient was placed in the supine position with the knee slightly flexed over a sterile roll positioned beneath the popliteal fossa; a tourniquet was applied at the root of the limb. Perioperative antibiotic prophylaxis consisted of cefazolin 2 g administered preoperatively and repeated at eight and 16 hours postoperatively. 

**Figure 1:**
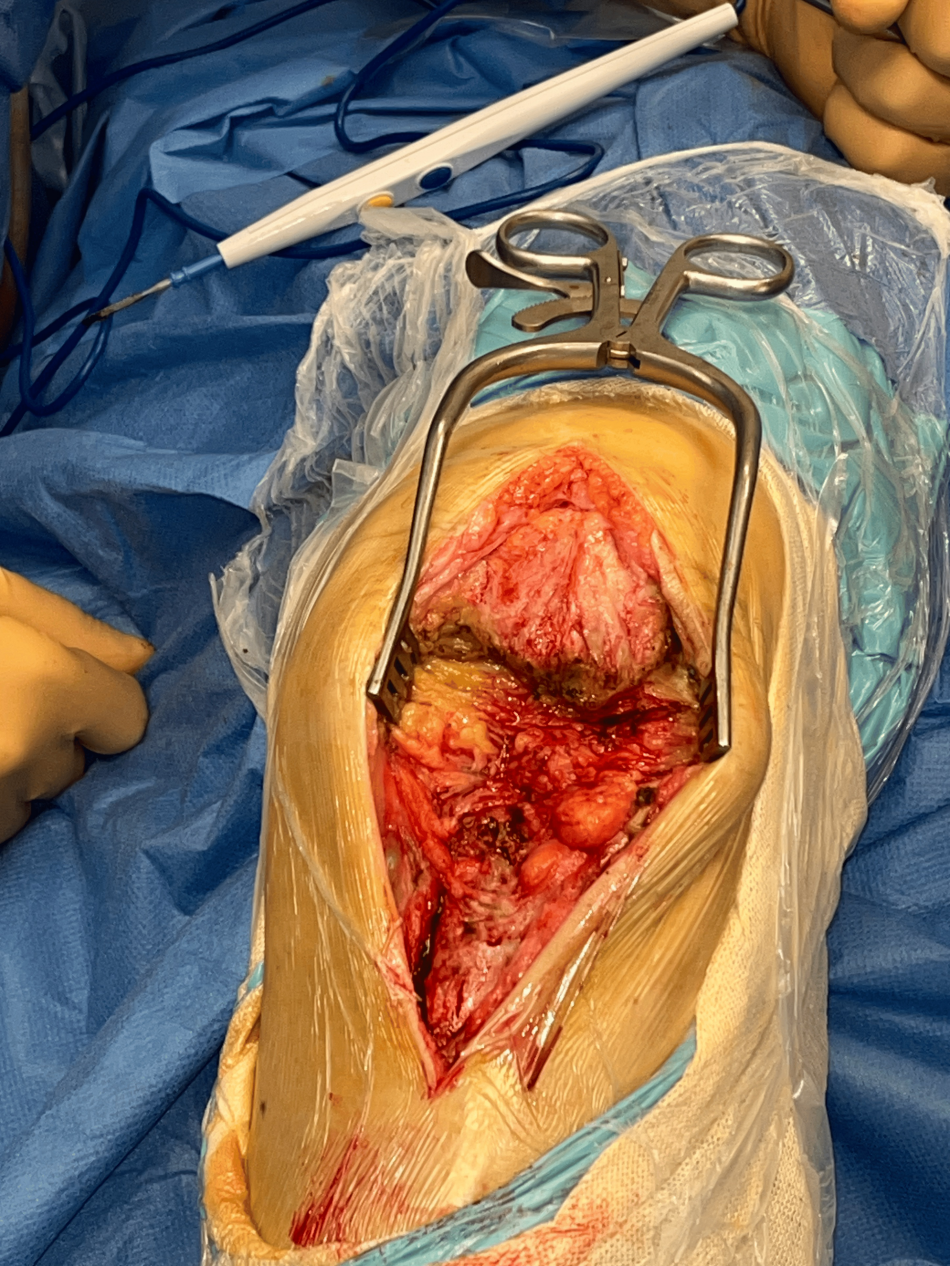
Intraoperative image showing a complete rupture of the PT.

A midline anterior incision was made, extending from the superior pole of the patella to the tibial tuberosity (TT). After incision of the subcutaneous layers, the residual tendon stumps were exposed; these appeared fibrotic, with an almost complete rupture of the PT, not suitable for direct repair (Figure 1).

**Figure 2:**
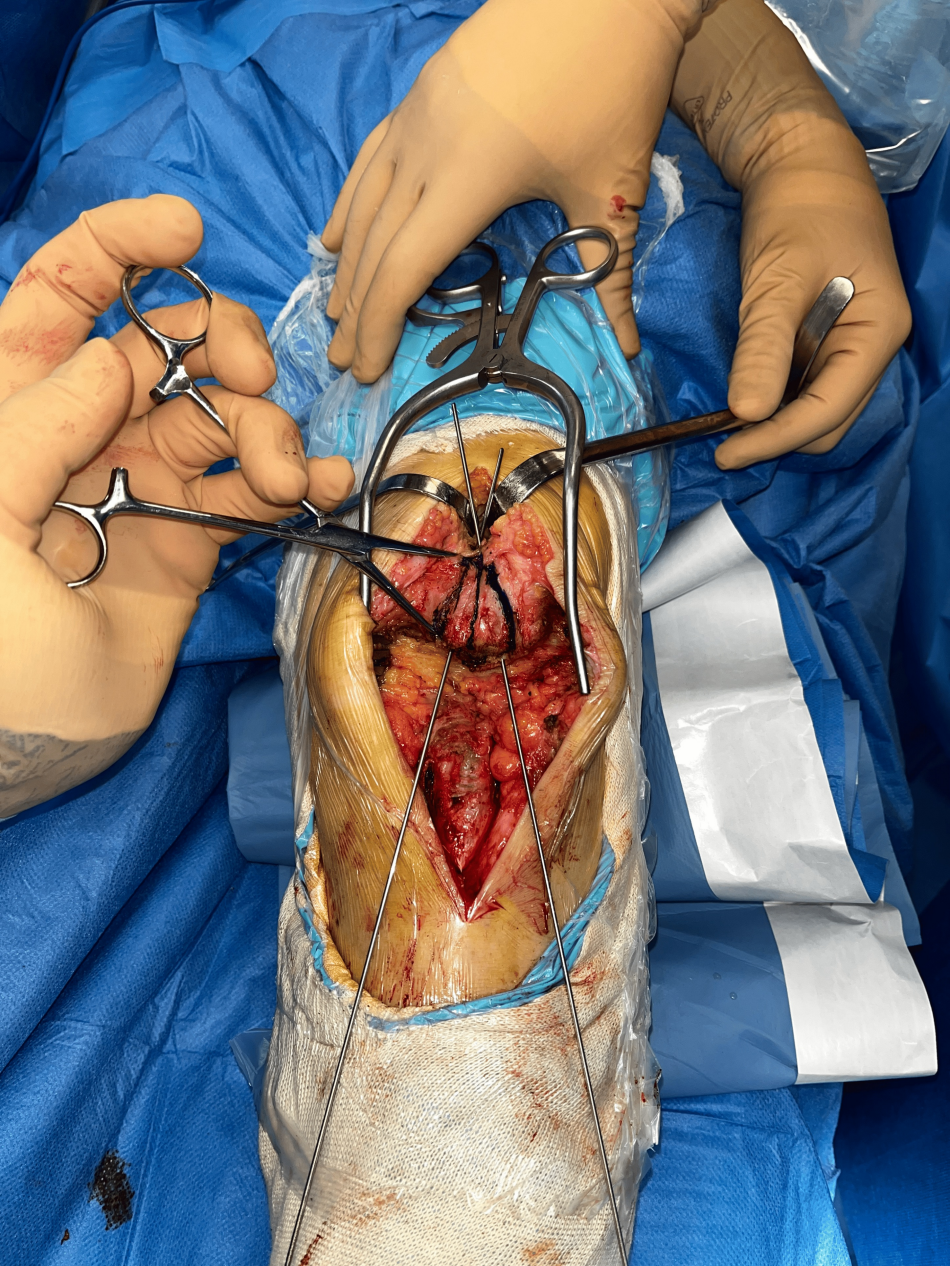
Converging patellar bone tunnels.

Two converging transosseous tunnels were then created in the patellar body, with proximal exit holes at the level of the superior pole, carefully avoiding the articular surface (Figure 2).

At the tibial level, a transverse tunnel was made approximately 1 cm distal to the TT. Given the potential osteopenic condition of the patella, smaller-diameter tunnels (about 4.5 mm) were preferred to reduce the risk of iatrogenic fracture, while the tibial tunnel was slightly larger (approximately 5 mm) to accommodate the graft and ensure secure fixation.

A peroneus longus tendon allograft was used, obtained from the Musculoskeletal Tissue Bank (BMTS) of the Lazio Region, Italy. The graft was processed and preserved according to institutional and national standards, including low-dose gamma irradiation and storage at -80°C until use. After preparation, the tendon measured approximately 24 cm in length and 5-6 mm in diameter. The graft was tubulized at the ends according to the “Roman sandal” technique and prepared on the back table with locking sutures using FiberWire No. 2 to facilitate passage through the bone tunnels and provide adequate tensioning.

**Figure 3:**
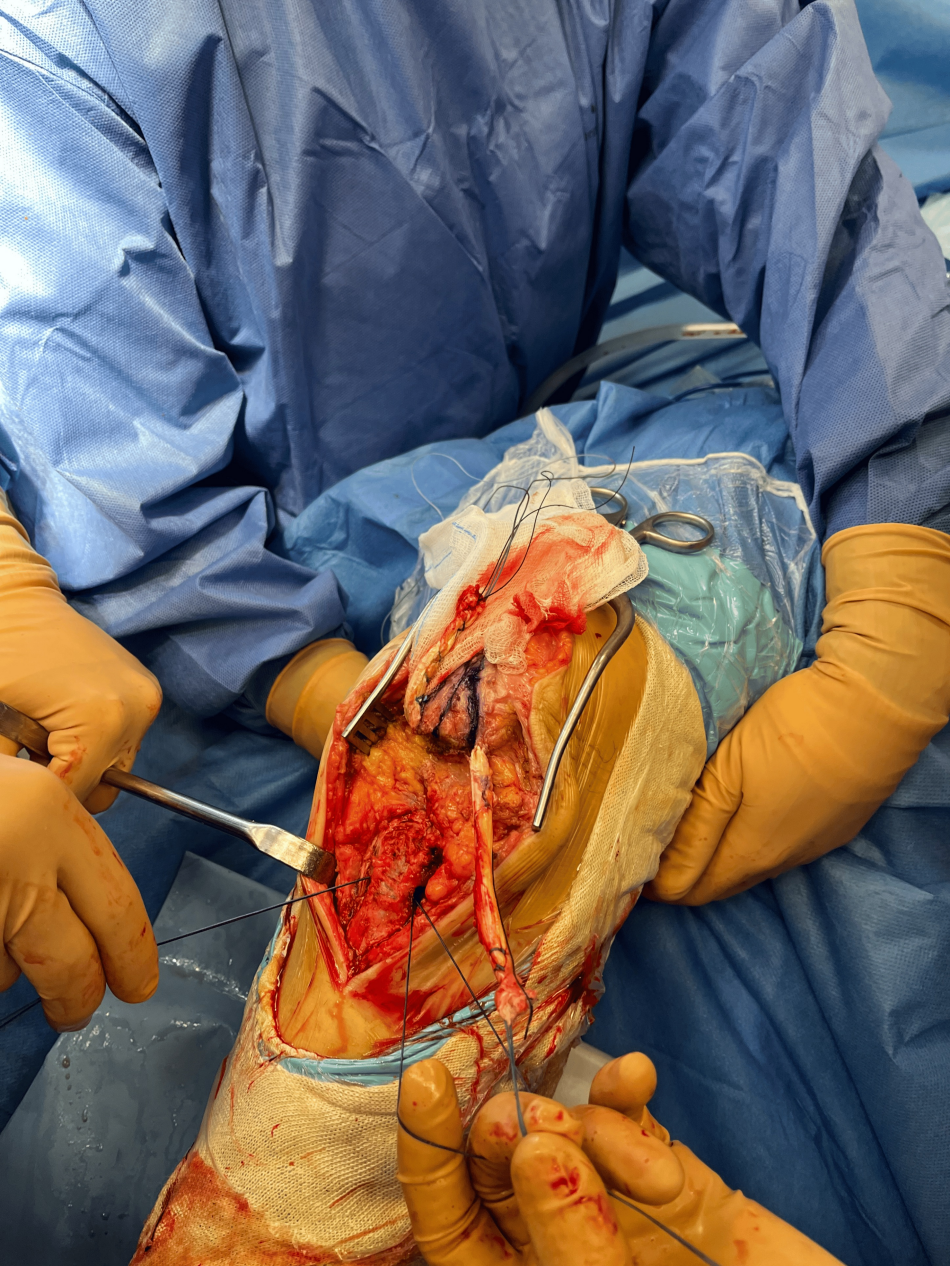
Passage of the allograft with shuttle sutures: the graft, already passed through the patellar tunnels, is positioned for transport through the tibial tunnel.

The distance between the distal pole of the patella and the native PT insertion was measured to assess patellar height. At this stage, the tourniquet was released to allow full quadriceps excursion and ensure accurate evaluation of patellar positioning. The prepared graft was then passed through the converging patellar tunnels and subsequently through the transverse tibial tunnel using shuttle sutures (Figure 3).

**Figure 4:**
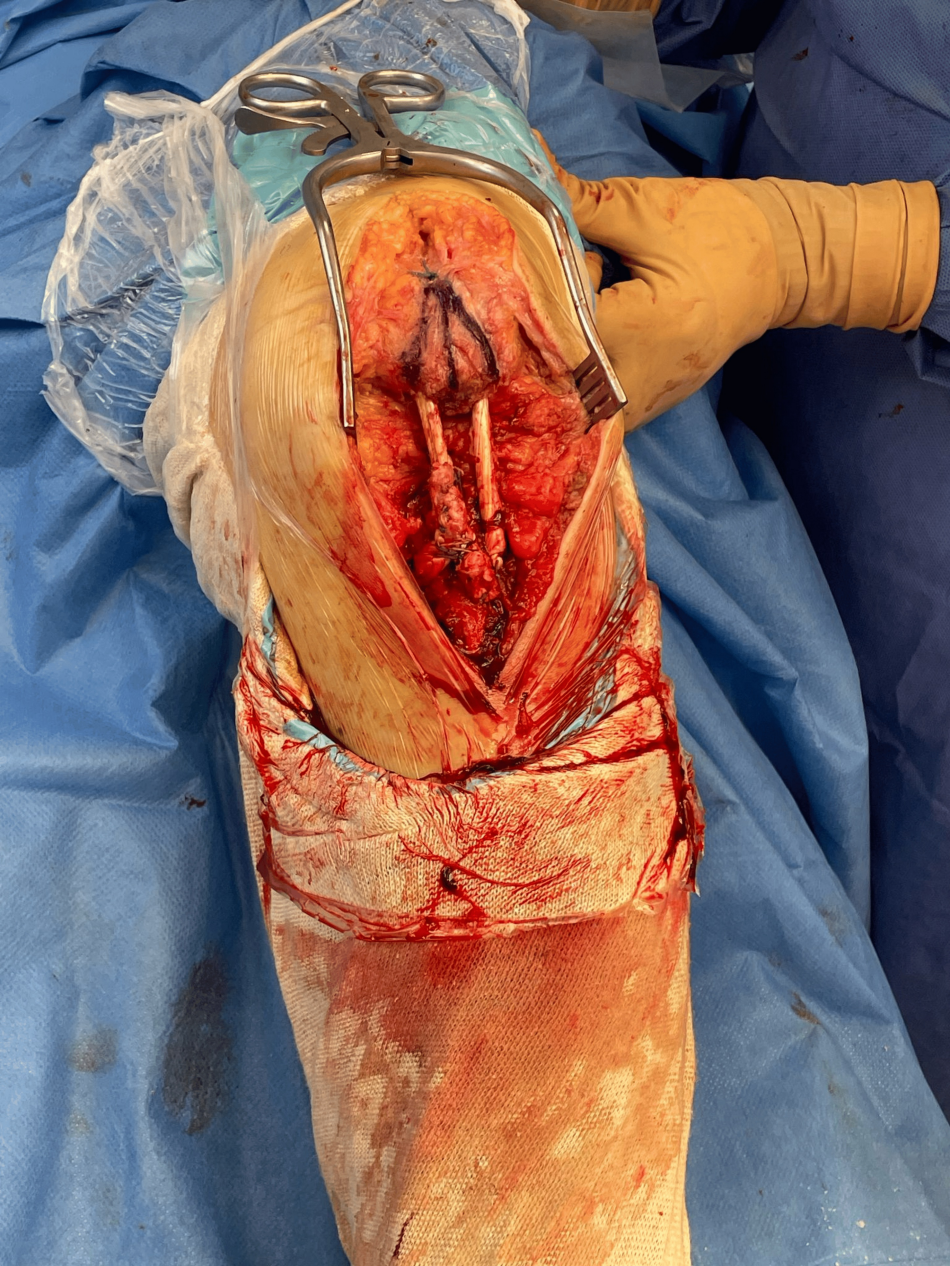
Final appearance of PT reconstruction with peroneus longus allograft: the graft is fixed proximally within the patellar tunnels and distally to the tibial tunnel with an anchor.

The proximal end of the allograft was sutured onto itself, while the distal end was fixed to the TT using a threaded anchor (Corkscrew, Arthrex®, Naples, FL) with preloaded high-strength nonabsorbable sutures, providing stable distal fixation (Figure 4).

Tensioning was verified with the knee positioned at 30° of flexion, confirming restoration of patellar height and correct alignment of the quadriceps line of pull.

Layered closure of the subcutaneous tissues and skin was performed according to the standard technique. Appropriate restoration of patellar height was confirmed intraoperatively by lateral knee fluoroscopy.

Postoperatively, the knee was immobilized in a hinged brace locked in full extension for four weeks. From the second postoperative week, assisted passive mobilization within a controlled range was allowed, while active mobilization and strengthening exercises were progressively introduced starting at week six. Return to full functional activity was expected between the ninth and 12th months.

Six months after surgery, the patient showed a clear functional recovery. Knee range of motion was -5° in extension and 110° in flexion; extensor strength, assessed by the MRC scale (Medical Research Council scale for muscle strength) [9], had improved from 0/5 preoperatively to 3/5. Pain levels, measured with the visual analog scale (VAS) [10], had decreased from 7 to 4. Functional scores also demonstrated significant improvement: the Knee Society Score (KSS) [11] increased from 45 to 65, and the Tegner Activity Level Scale [12] from 1 (restricted to household ambulation) to 2-3 (assisted walking and light activities). At the 12-month follow-up, reconstruction stability was confirmed, with no complications and further functional gains compared to the preoperative condition. A mild extension deficit and reduced extensor strength persisted, most likely related to the underlying neuromuscular condition (post-poliomyelitis sequelae) (Table 1). 

**Table 1:** Clinical results KSS = Knee Society Score (range 0-100); MRC = Medical Research Council Scale (range 0-5); ROM = Range of Motion (°); VAS = Visual Analog Scale (range 0-10); Tegner Activity Level Scale (range 0-10).

Outcome measure	Preoperative	Six months	12 months
Knee ROM (°)	-20° extension/80° flexion	-5° extension/110° flexion	-5° extension/115° flexion
Extensor muscle strength (MRC scale)	0/5	3/5	3/5
Pain (VAS)	7	4	3
KSS score	45	65	70
Tegner Activity Level Scale	1 (household ambulation only)	2-3 (assisted walking, light activities)	2-3 (stable, light activities)
Complications	None	None	None

Postoperative lateral knee radiographs demonstrated appropriate restoration and maintenance of patellar height, with an Insall-Salvati ratio of 0.85 [13], consistent with a normal range. 

## Discussion

Hamstring autografts remain widely used due to their biological potential and relative ease of harvest, and chronic PT ruptures often present tendon retraction, poor tissue quality, and established functional deficits, making direct repair impracticable, particularly in elderly patients or those with neuromuscular comorbidities [4,8].

Several reconstruction techniques have been described in the literature, using either autografts (semitendinosus, gracilis, quadriceps) or allografts (Achilles tendon, anterior tibialis, hamstrings) [14]. Autografts provide good biological integration but are associated with donor-site morbidity and longer operative times. Allografts, on the other hand, minimize local morbidity and allow for more versatile graft shaping, though at the expense of higher costs and a theoretical risk of immune response.

In our case, a peroneus longus tendon allograft was used, which proved suitable in terms of length, mechanical strength, and ease of tubulization. Compared with the Achilles tendon, which is more commonly employed in this setting, the peroneus longus offers easier adaptation to bone tunnels and requires a smaller tissue volume [15]. Recent case reports and case series have confirmed the effectiveness of this graft, reporting favorable clinical and functional outcomes in both post-traumatic reconstructions and post-TKA settings [14,15].

Several potential pitfalls must be considered during reconstruction. Incorrect restoration of patellar height, with graft tensioning in the presence of patella alta or baja, may lead to functional limitations and anterior knee pain. Improper placement of patellar tunnels also represents a significant risk: tunnels too close to the articular surface increase the likelihood of fracture or secondary osteoarthritis, while overly wide or convergent tunnels in osteopenic bone may cause iatrogenic patellar fractures. Another potential issue is graft laxity, which can occur if tensioning is not performed with the knee flexed at 30°. Inadequate distal fixation, due to weak anchorage at the TT, may also result in early graft failure. In patients with neuromuscular deficits, a cautious approach is required, as unrealistic functional expectations may lead to misinterpreting a clinically acceptable result as a failure. Additionally, in individuals with previous scarring or poor skin quality, an extended incision may compromise local vascularity.

Certain technical measures may reduce these risks. Intraoperative fluoroscopic assessment of patellar height on a lateral view, compared with the contralateral side, allows accurate restoration of positioning. Graft tensioning should be performed at 30° of flexion while simultaneously verifying the quadriceps trajectory. To minimize the risk of patellar fracture, the use of small-diameter tunnels (3-4 mm) is recommended. In cases of significant quadriceps retraction, a V-Y lengthening may be required to prevent patella alta. Preparing the allograft in tubulized form, according to the “Roman sandal” technique, facilitates passage and improves tensile strength. It is also essential to verify proper anchor suture function before final fixation. From a rehabilitation perspective, an initial immobilization period should be followed by early introduction of passive motion to reduce the risk of stiffness and adhesions. In patients with poliomyelitis or neurological deficits, management of expectations remains critical: the realistic goal is not restoration of normal extensor strength, but achievement of a functional level compatible with daily activities.

Another important consideration is the potential for bone tunnel widening, a complication well documented in anterior cruciate ligament (ACL) reconstructions, particularly with hamstring grafts, which share anatomical and biomechanical similarities with the peroneus longus. The phenomenon is driven by biological factors such as bone resorption mediated by inflammatory cells and metalloproteinases, and by mechanical factors, particularly the windshield-wiper and bungee-cord effects, which generate graft micromotion within the tunnel. Several studies have reported that hamstring grafts are at greater risk of tunnel widening compared with bone-patellar tendon-bone (BPTB) grafts [12,14]. Clatworthy et al. [16] observed a greater mean increase in tunnel diameter with hamstring grafts than with PT grafts, while Fauno and Kaalund [17] demonstrated that fixation type influences the extent of widening, with suspensory devices (e.g., Endobutton, TightRope) associated with greater tunnel enlargement than interference screw fixation. Giorgio et al. [18] confirmed the correlation between fixation system elasticity and tunnel widening. To date, no data are available on tunnel widening in PT reconstructions, with existing evidence almost exclusively derived from ACL reconstructions using hamstring grafts. Therefore, by biomechanical analogy, such data may serve as an indirect reference for predicting the behavior of the peroneus longus tendon.

Allografts such as the peroneus longus offer several relevant advantages. The absence of donor-site morbidity is particularly important in frail patients or those with neuromuscular deficits [15,19]. Furthermore, the availability of grafts with adequate length and diameter makes them suitable even in chronic cases with tendon retraction [15,19]. Additional advantages include high mechanical strength and ease of tubulization, which facilitate application in transosseous techniques [15], as well as demonstrated versatility in both post-traumatic reconstructions and post-TKA contexts [14,19]. Nevertheless, some limitations remain. Availability is more restricted compared with Achilles tendon allografts, and costs are generally higher due to reliance on tissue banking. A theoretical risk of immune response or infectious disease transmission persists, though this risk has been significantly reduced by modern sterilization methods [14]. Finally, the current scientific evidence is limited, consisting mainly of case reports and small series, with a lack of large prospective studies [15,19].

A particular feature of our patient was the history of poliomyelitis, a rare but still occasionally encountered condition. In such cases, the realistic goal is not complete restoration of extensor strength, but rather pain reduction and recovery of sufficient active extension to perform daily activities. Specific data for patients with poliomyelitis are scarce; however, biologic techniques using allografts appear to provide satisfactory results even in complex neuromuscular contexts [20].

Our results, with the absence of complications and functional recovery consistent with pre-existing conditions, align with the literature regarding the efficacy of the peroneus longus tendon. The described technique represents a valid option for chronic PT rupture when primary repair is not feasible, and donor-site morbidity from autograft harvest is to be avoided.

This study has inevitable limitations: it is a single case with a relatively short follow-up, which does not allow definitive conclusions about long-term efficacy. However, the detailed description of the procedure and the preliminary functional outcomes make the technique potentially reproducible in selected settings.

## Conclusions

In this case of chronic subtotal PT rupture in a post-polio patient, reconstruction with a peroneus longus tendon allograft resulted in satisfactory pain relief, restoration of active extension, and stable functional recovery at 12 months. The technique provides adequate mechanical strength while avoiding donor-site morbidity, making it a suitable option in neuromuscular patients with compromised local tissue quality.

Future research should include biomechanical evaluations and comparative studies with Achilles tendon allografts to better define the long-term role of peroneus longus allografts in chronic PT reconstruction.
